# Highlights lecture EANM 2016: “Embracing molecular imaging and multi-modal imaging: a smart move for nuclear medicine towards personalized medicine”

**DOI:** 10.1007/s00259-017-3704-6

**Published:** 2017-06-08

**Authors:** Eric O. Aboagye, Françoise Kraeber-Bodéré

**Affiliations:** 1 Cancer Imaging Centre, Department of Surgery & Cancer, Imperial College London, London, UK; 2 Nuclear Medicine, Hôtel Dieu University Hospital, 1 place Alexis Ricordeau, Nantes, 44093 France; 3 CRCINA, Inserm U1232, Nantes, France; 4 Nuclear Medicine, ICO Cancer Center, Saint-Herblain, France

**Keywords:** Highlights lecture, EANM, Personalized medicine, Oncology, Cardiology, Neurosciences

## Abstract

The 2016 EANM Congress took place in Barcelona, Spain, from 15 to 19 October under the leadership of Prof. Wim Oyen, chair of the EANM Scientific Committee. With more than 6,000 participants, this congress was the most important European event in nuclear medicine, bringing together a multidisciplinary community involved in the different fields of nuclear medicine. There were over 600 oral and 1,200 poster or e-Poster presentations with an overwhelming focus on development and application of imaging for personalized care, which is timely for the community. Beyond FDG PET, major highlights included progress in the use of PSMA and SSTR receptor-targeted radiopharmaceuticals and associated theranostics in oncology. Innovations in radiopharmaceuticals for imaging pathologies of the brain and cardiovascular system, as well as infection and inflammation, were also highlighted. In the areas of physics and instrumentation, multimodality imaging and radiomics were highlighted as promising areas of research.

## Introduction

The choice of title for the 2016 EANM highlights lecture resonates with the steady rise in new imaging methods for personalized medicine. As seen at the conference, the breadth of imaging methods being developed and/or applied to personalize therapy is remarkable, ranging from molecular characterization to direct treatment pathways and choice of theranostics, to the promise of radiomics and other biomathematical approaches for predicting therapy response and patient outcome. As a community, we are paralleling the explosion in different facets of medicine including immunotherapy and radionuclide therapy through implementation of multimodal molecular imaging diagnostic approaches that will not only make better use of these powerful therapies but also, in the future, help us to better judge therapy resistance and how to manage it. The scientific reports submitted to this congress – 2,266 in total – highlight the ability to design new vectors to navigate the body’s defences and home in on specific pathologies including cancer, dementia, cardiovascular disease, infection and more. These reports show that our community is changing how medicine is practised and will be practised in the future in terms of who should get what therapy, whose therapy ought to be changed, and how best to deliver therapy.

The era of personalized or stratified medicine began a few years ago, especially in oncology but also in neurology and cardiology. Over the past few years, nuclear medicine has undergone impressive growth, with the development of PET, especially using ^18^F-fluorodeoxyglucose (^18^F-FDG), but also innovative radiopharmaceuticals. These developments are ripe for personalized medicine including ‘smart’ diagnostic tools with high sensitivity and specificity, baseline prognostic imaging or therapy evaluation tools, and predictive imaging (theranostics and companion diagnostics) approaches using novel radiopharmaceuticals targeting relevant biomarkers to allow selection of patients for conventional and radionuclide-targeted therapies.

The scientific programme of the EANM congress in Barcelona illustrates the considerable growth of nuclear medicine with 1,892 scientific communications in the field of oncology (31%), the molecules to man (M2M) pathway including translational molecular Imaging, radiopharmacy, radiochemistry and drug development (21%), radionuclide therapy and dosimetry (12%), physics, instrumentation and data analysis (10%), cardiology (7%) and neuroscience (7%). In oncology, prostate cancer, neuroendocrine tumours (NETs), breast cancer, brain tumours and haematological diseases were the tumours of most interest for nuclear medicine development. From more than 200 excellent presentations received for highlights, only 50 were finally included in the lecture, because of the limited time available and redundancy between presentations. However, we congratulate all authors who provided slides for their excellent contributions.

## Discovery science

**Fig. 1:**
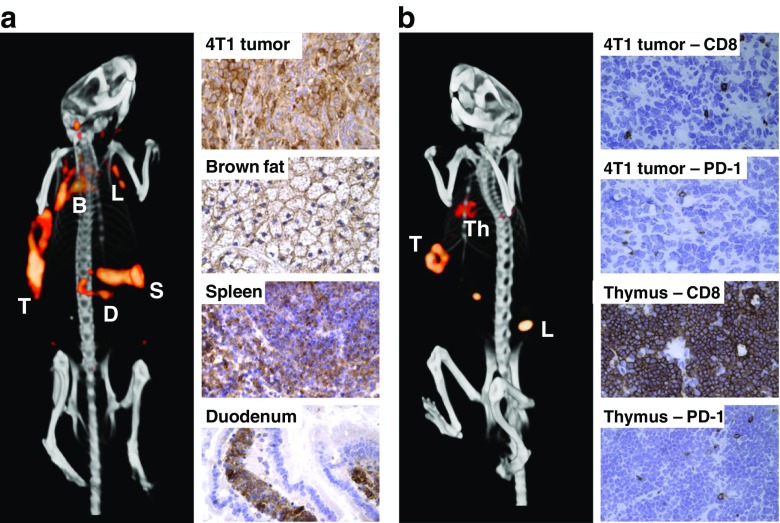
**a** Imaging with anti-PD-L1 antibodies shows uptake in the tumour, spleen, duodenum, brown fat, and lymph nodes. **b** Imaging with anti-PD-1 antibodies shows uptake in the tumour, lymph nodes and thymus. *B* Brown fat, *D* Duodenum, *L* Lymph nodes. *S* Spleen, *T* Tumour, *Th* Thymus

We received a large number of abstracts dealing with innovative radiopharmaceuticals with new delivery systems and radionuclides. The number of cell-surface biomarker imaging probes continues to increase. Based on the negative prognostic role of epidermal growth factor receptor (EGFR) in head and neck squamous cell carcinoma [1, 2], Burley et al. from London, UK, developed a PET tracer for imaging EGFR by conjugating the long-lived isotope ^89^Zr to an Affibody specific for EGFR via the chelator deferoxamine (^89^Zr-DFO-Affi_EGFR:03115_) [3]. PET/CT with this tracer showed EGFR-specific and sensitive tumour localization in xenografted mice. Tumour heterogeneity was demonstrated by immunohistochemistry and autoradiography. In addition to EGFR, another target set linked to major drug development programmes is the immune checkpoint PD-1 expressed on T cells, and its ligand PD-L1, expressed on tumour and antigen-presenting cells [4]. Heskamp et al. from Nijmegen, The Netherlands, radiolabelled both anti-PD-1 and anti-PD-L1 antibodies with ^111^In to image PD-1 and PD-L1 expression, respectively, in syngeneic murine tumour models [5]. In tumour, spleen, duodenum, brown fat, lymph nodes and thymus, ^111^In-anti-PD-1 and ^111^In-anti-PD-L1 uptake, detected by SPECT/CT 3 days after injection, was associated with expression of the respective organ proteins by immunohistochemistry, supporting the role of these radiolabelled antibodies in pretreatment imaging and therapy monitoring (Fig. 1).

**Fig. 2:**
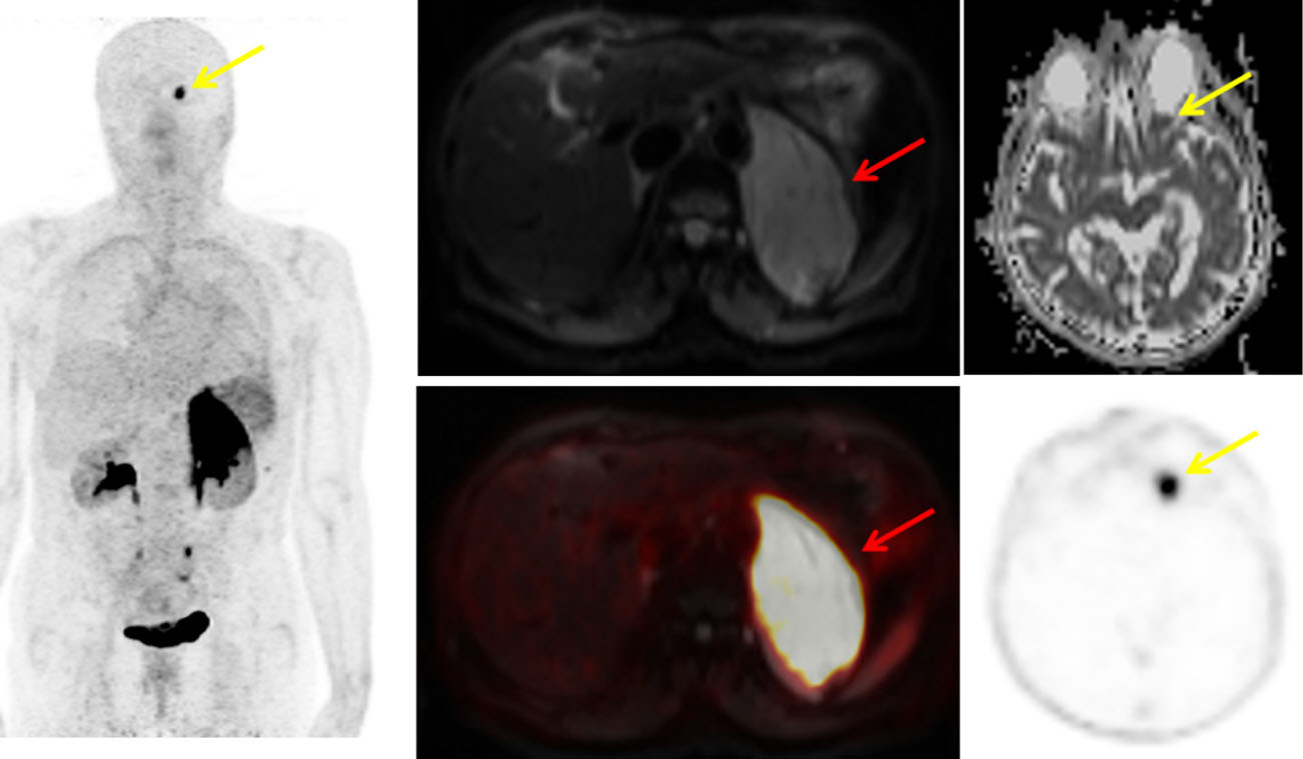
^68^Ga-Pentixafor PET in MALT lymphoma. A 75-year-old woman with biopsy-proven MALT lymphoma of the pararenal region. The known large MALT lymphoma shows high ^68^Ga-pentixafor uptake (SUVmax 25.4) and restricted diffusion on the diffusion-weighted images with b value 800 (*red arrows*). In addition an area of increased uptake on the PET images with restricted diffusion on the ADC map (*yellow arrows*) is evident, which proved to be MALT lymphoma of the orbit

**Fig. 3:**
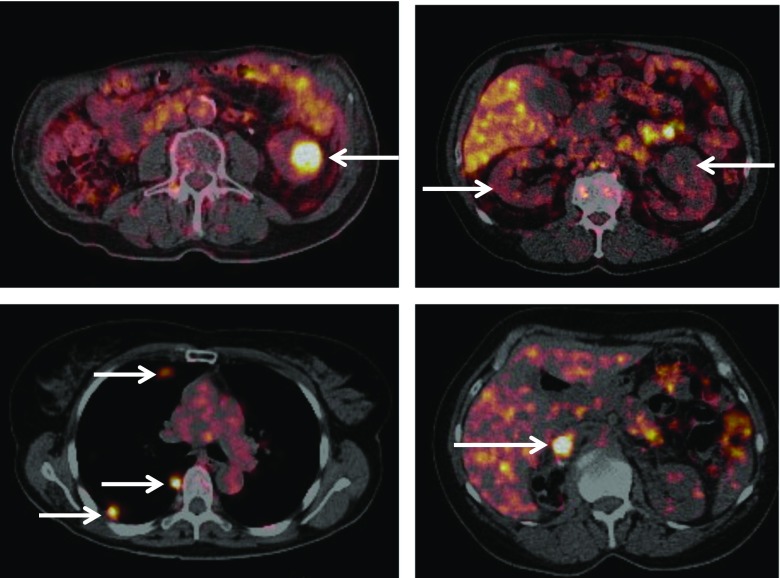
^89^Zr-Girentuximab PET imaging. *Top left*: A 76-year-old patient presenting with a Bosniak category 3 renal cyst in a solitary kidney and decreased renal function. ^89^Zr-Girentuximab PET/CT imaging indicated that it was a ccRCC, which was confirmed after partial nephrectomy. *Top right*: Patient presenting with bilateral growing renal masses. No uptake is seen in any of the lesions. Three biopsies confirmed that the masses were oncocytomas. *Bottom left*: Patient with a history of ccRCC who developed a large liver lesion and multiple pulmonary metastases. The ^89^Zr-girentuximab PET/CT image shows uptake in all lesions confirming metastatic ccRCC. *Bottom right*: Patient presenting with a 1-cm mass in the inferior caval vein 6 months after nephrectomy for ccRCC. The ^89^Zr-girentuximab PET/CT image shows high uptake in the mass and thrombectomy confirmed vital ccRCC

The CXCR4 and CAIX targets were also highlighted. Research on the chemokine receptor CXCR4 continues to gain momentum. Miranda et al. from Hull, UK, reported a new specific and stable configurationally restricted tetraazamacrocyclic CXCR4 antagonist, ^64^Cu-CB-bicyclam, suitable for PET imaging of CXCR4 with high affinity [6]. They demonstrated that tumour uptake was dependent on CXCR4 expression, and further showed self-blocking with cold tracer in liver but not with AMD3100 or AMD3465 – presumably due to higher affinity and residence time of the tracer. There were also reports of the first clinical tracer in this field, ^68^Ga-pentixafor [7]. Leisser et al. from Vienna, Austria, reported high contrast PET/MRI with this tracer in patients with the indolent MALT lymphoma, even when the lesion was undetectable by MRI [8] (Fig. 2). The clinical development of antibody vectors for CAIX was also highlighted. Hekman et al. from Nijmegen, reported the first-in-man study of the anti-CAIX antibody, girentuximab, labelled with ^89^Zr, in patients with renal cell carcinoma (RCC) [9]. Their study exemplifies how this PET/CT strategy could have a role in detecting or excluding primary and metastatic clear cell RCC (ccRCC) (Fig. 3). Furthermore, the observed association between PET and histology means that in a situation where MRI was not able to differentiate between blood thrombus and vital tumour thrombus, PET/CT showed high uptake of ^89^Zr-girentuximab in the mass, and thrombectomy confirmed vital ccRCC. Lastly, Iagaru et al. from Stanford, USA, presented work on ^68^Ga-RM2 PET/MRI measuring gastrin-releasing peptide receptor, and demonstrated its potential utility in the specific detection of biochemically recurrent prostate, an area that has seen several new tracers recently approved for human use, with PET detecting more lesions than MRI [10].

The overarching direction of travel in oncology discovery science appears to be design and validation of highly specific probes, with the potential to transform our understanding of cell surface and extracellular matrix molecular pathology and to provide opportunities for detecting and treating disease. While the majority of radiopharmaceutical discovery science was in oncology there were also reports of innovative probes in neuroscience, cardiovascular disease, infection/inflammation and other fields. Three new brain targets for PET imaging were highlighted including β-secretase (a key enzyme in β-amyloid deposition) [11], the cyclic adenosine monophosphate regulator phosphodiesterase 4 [12], and α-synuclein [13]. Takano et al. from Stockholm, Sweden, reported the radiolabelling and initial evaluation of a novel β-secretase-selective PET radioligand, ^18^F-PF-06684511, with favourable binding kinetics in the brain of nonhuman primates [14]. The authors proposed that the probe could be a valuable PET imaging translational tool for elucidating the disease mechanism of Alzheimer’s disease and for facilitating clinical development of β-secretase inhibitors as a potential treatment for Alzheimer’s disease.

**Fig. 4:**
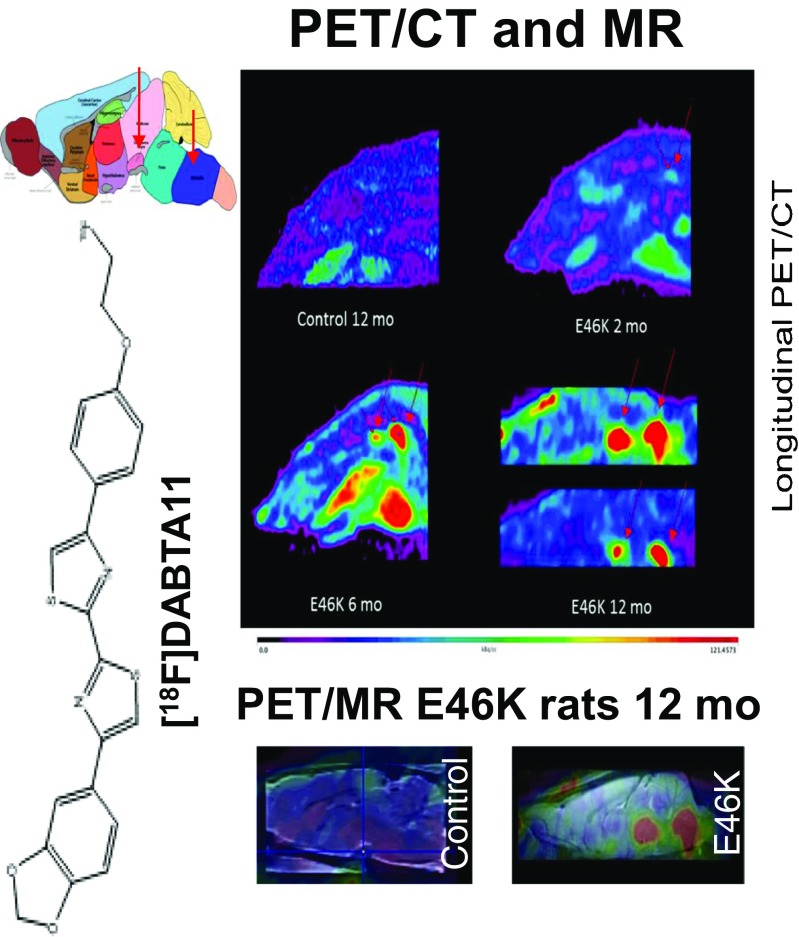
[^18^F]DABTA-11 PET images in E46K rats show accumulation of the tracer in the medulla oblongata. The accumulation is apparent even at 2 months of age and is more prominent at 6 and 12 months of age with detectable uptake in the substantia nigra. PET/MRI and rat brain atlas confirm the regional uptake of tracer

 The development of ^18^F-PF-06445974, a novel PDE4D-sparing PDE4 PET radioligand was reported by Chen et al. from Cambridge, USA [15]. The tracer demonstrated favourable brain binding kinetics in nonhuman primates with high tissue distribution and up to 94% blockade of ligand occupancy. This study precedes the development of new inhibitors for treating diseases such as psychosis without the side effect of emesis. From a library of compounds with selectivity (>120-fold) to α-synuclein relative to Aβ and tau, Yousefi et al. from Munich identified a high-affinity tracer, ^18^F-DABTA-11, for visualization of α-synucleinopathies by PET [16]. ^18^F-DABTA-11 PET/MRI imaging of NTac:SD-Tg(SNCA*E46K) genetically modified mice confirmed regional uptake of the tracer, as verified by immunohistochemistry (Fig. 4). The tracer holds promise for the early diagnosis of α-synucleinopathies including Parkinson’s disease and dementia with Lewy bodies.

Two tracers in cardiovascular disease targeting fatty acid metabolism and mannose receptor expression were also highlighted. Strauss et al. from New York, USA, reported a phase II study of a novel fatty acid analogue, ^18^F-FCPHA, for PET imaging of cardiac metabolism [17]. There were no serious adverse events and seven adverse events. High diagnostic-quality images of the heart were obtained at rest and stress (exercise or dipyridamole) 30 min after tracer injection. Varasteh et al. from Munich described the use of ^111^In-tilmanocept for imaging the mannose receptor by SPECT/CT [18]. The authors demonstrated specific imaging of the mannose receptor in ApoE-KO mice. Histology showed the presence of mannose receptor-positive M3/84 (Mac3 macrophages surface antigen) and fibrous/fibroatheromatous plaques in parts of the aorta, and these were detected macroscopically by SPECT/CT and by ex vivo imaging. Molecular imaging in non-oncology areas is clearly expanding and in some cases ahead of drug development programmes, and thus promises to be highly valuable in the clinical implementation of the drugs in such programmes.

New targetry, synthetic routes and automation permit improved and safe production of radiopharmaceuticals. In spite of the value of instrumentation, we were prevented by the constraint of time from elaborating the various presentations at this conference. One area worth highlighting involves developments around scandium radioisotopes [19, 20]. Bilewicz et al. from Warsaw presented work on cyclotron production of the theranostic pair ^43^Sc (β^+^ emitter for PET; *t*_1/2_ 3.89 h) and ^47^Sc (β^−^ emitter for radionuclide therapy) [21]. They were able to produce ^43^Sc in ^42^Ca(d,n)^43^Sc with no radionuclide impurities, and ^47^Sc in ^48^Ca(p,2n)^47^Sc nuclear reactions with low radionuclide impurity profile (<10% ^48^Sc). The scandium radionuclide pair could be a cyclotron alternative to ^68^Ga in theranostic applications. Other applications for PET imaging were reported at the conference.

## PET/MRI: useful for science but what about clinical practice?

PET/MRI represents a significant advance in multimodality molecular imaging [22] and more abstracts were received for the 2016 congress than for previous congresses, with 16 presentations considered for the Highlights lecture, in particular studies addressing the impact of PET/MRI in clinical practice. As expected, brain tumour evaluation is an important issue for this multimodality imaging. Grech-Sollars et al. from Imperial College London presented a prospective pilot study which did not use a PET/MRI system but addressed a relevant question for PET/MRI [23]. Choline is a biomarker of interest in glioma that is related to cell membrane turnover and tumour aggressiveness, and previous choline PET studies have shown differential uptake between benign and malignant tumours. Thus, the relationship between choline metabolism detected in vivo using MR spectroscopy (MRS) and PET, and tissue markers of choline synthesis and glioma proliferation were investigated in 14 patients with suspected primary glioma undergoing multimodal 3-T MRI (including multivoxel MRS) and dynamic 45-min list-mode ^18^F-fluoromethylcholine (^18^F-FMC) PET/CT. The ratio of the tumour SUVmax to the contralateral white matter SUVmean (tumour to background ratio, TBR) was determined. All tumours showed increased tracer uptake with spatial concordance between PET and MRS. Higher TBR values were observed in WHO grade IV tumours than in WHO grades I–III tumours on ^18^F-FMC PET/CT (mean 25.5 versus 8.2; *p* < 0.05) and higher Cho/Cr ratios were observed in WHO grades III/IV tumours than in grade I/II tumours on MRS (*p* < 0.05), suggesting the relevance of multimodal imaging for glioma grading.

 We know that the attenuation correction (AC) method in PET/MRI is debatable, and Ladefoged et al. from the University of Copenhagen reported the new AC method RESOLUTE [24] in 68 fluoroethyltyrosine (^18^F-FET) PET/MRI examinations [25]. The use of RESOLUTE enabled measurement patient-specific bone density. Using RESOLUTE, improved PET recovery (−2%) was obtained over vendor-provided UTE (−7%) and Dixon (−15%) methods. The Tmax/B ratio is a metric used to distinguish tumour tissue from reactive tissue, and the improved PET accuracy resulted in Tmax/B ratios very close to those obtained with CT-AC. The improvement in accuracy obviously also affected tumour delineation, resulting in tumour volumes that were very close to the volumes found using CT-AC.

**Fig. 5:**
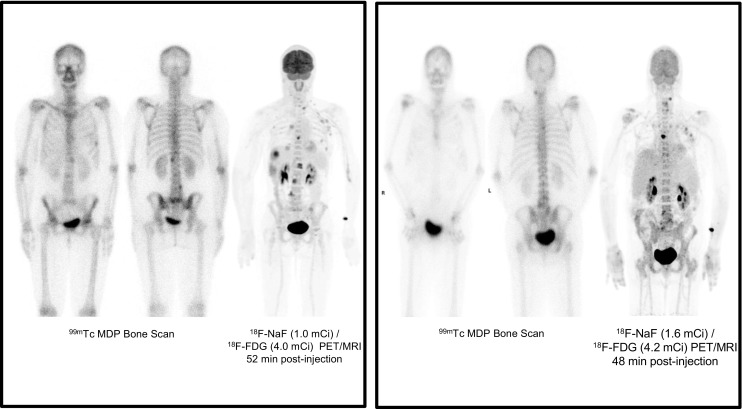
^18^F-NaF/^18^F-FDG PET/MRI in breast and prostate cancer. PET/MRI image quality was diagnostic despite the marked reduction in the administered activities. PET/MRI was superior to ^99m^Tc-MDP scintigraphy for evaluation of skeletal disease extent and detected extraskeletal disease

**Fig. 6:**
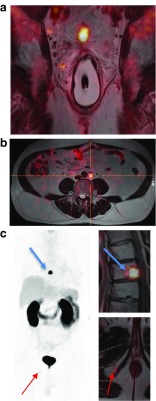
^68^Ga-PSMA-HBED-CC/^18^F-fluorocholine PET/MRI in prostate cancer: **a** locoregional lymph nodes; **b** paraaortic lymph node; **c** bone metastasis

We also received two interesting presentations reporting clinical data using dual tracers for PET/MRI in prostate and breast cancers. Sonni et al. from Stanford University reported the results of a prospective study assessing the combined use of ^18^F-NaF/^18^F-FDG and TOF simultaneous PET/MRI imaging in 11 women with breast cancer and 26 men with prostate cancer, in comparison with ^99m^Tc-MDP [26]. ^18^F-NaF (0.7–2.2 mCi, mean 1.1 mCi) and ^18^F-FDG (3.6–5.5 mCi, mean 4.1 mCi) were subsequently injected from separate syringes. The whole-body MRI protocol consisted of T2-weighted, DWI, and contrast-enhanced T1-weighted imaging. It is perhaps not surprising that ^18^F-NaF/^18^F-FDG PET/MRI was superior to ^99m^Tc-MDP scintigraphy, with higher lesion detection performance, even in bone (Fig. 5). The dual tracer PET/MRI procedure appears interesting, with good PET image quality despite a marked reduction in the administered activities of radiopharmaceuticals (80% less for ^18^F-NaF and 67% less for ^18^F-FDG compared with standard protocols) allowing a significant reduction in radiation exposure. Moreover, Hartenbach et al. from the Medical University of Vienna reported the clinical impact and staging performance of hybrid multiparametric ^68^Ga-PSMA-HBED-CC/^18^F-fluorocholine PET/MRI (dtPET/MRI) in 87 patients with biopsy-proven prostate cancer scheduled for radical prostatectomy included in a prospective clinical trial (NCT02659527) [27]. As compared to MSKCC prediction nomogram, dtPET/MRI using a dynamic protocol provided additional highly accurate information on TNM staging, leading to a treatment change in 26 of 87 patients (30%) (Fig. 6).

**Fig. 7:**
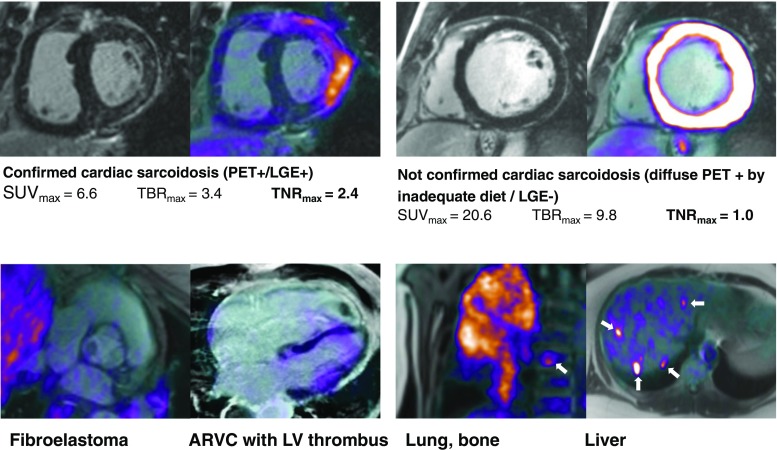
^18^F-FDG PET/MRI images in patients with suspected cardiac sarcoidosis. *ARVC* Arrhythmogenic right ventricular cardiomyopathy, *LGE* Late gadolinium enhancement, *LV* Left ventricle

PET/MRI is not only useful in oncology. Abgral et al. from the University Hospital of Brest, in collaboration with teams at the University of Edinburgh and the Icahn School of Medicine at Mount Sinai, investigated the role of ^18^F-FDG PET/MRI in the detection of cardiac sarcoidosis (CS), which is a diagnostic challenge [28]. ^18^F-FDG is a marker of inflammatory disease, and MRI with late gadolinium enhancement (LGE) reveals the pattern of myocardial injury. In this study, PET data were reconstructed using a Dixon MRI AC map. Of 25 patients prospectively enrolled, 8 were considered CS-positive and 17 CS-negative by a consensus of clinical experts. Interestingly, while there was no statistically significant difference in terms of SUVmax (*p* = 0.266), T2 mapping value (*p* = 0.320), target-to-blood pool ratios (TBRmax, *p* = 0.239; TBRmean, *p* = 0.248) between CS-positive and CS-negative patients, the mean maximum target-to-negative myocardium LGE ratios (TNRmax) were 1.68 ± 0.41 and 1.09 ± 0.08 in CS-positive and CS-negative patients, respectively (*p* < 0.0001). Moreover, two patients with previously unknown sarcoid involvement (bone, liver) were identified and PET/MRI identified an alternative cause for cardiac symptoms in six CS-negative patients (Fig. 7).

## PSMA and somatostatin receptor imaging and therapy: high throughput developments

PSMA targeting using radiolabelled ligands was a major topic at the EANM 2016 congress with around 50 communications initially considered for Highlights. These compounds based on lysine-glutamate-urea derivatives, first developed as inhibitors of the enzymatic activity of PSMA, have been labelled by a variety of radionuclides for PET imaging and radionuclide therapy for theranostic approaches in prostate cancer patients. A variety of closely related compounds are under investigation [29]. Retrospective studies have suggested a high sensitivity of PET using ^68^Ga-PSMA ligands in early restaging of prostate cancer but, as with other PET tracers, their value in primary tumour staging is yet to be determined, especially in patients with high-risk disease [30, 31]. Moreover, large prospective studies are required to confirm their imaging performance and clinical impact. Other PET emitters with longer half-lives such as ^64^Cu could also be interesting in allowing delayed imaging and quantification, and also centralized production. For therapeutic applications, despite toxicities that need to be better controlled, PSMA radioligand therapy (PRLT) is an original systemic approach to the treatment in patients with relapsing metastatic disease, and promising efficacy has been reported with ^177^Lu [32, 33], as well as with alpha-emitters [34], with preclinical studies suggesting a potentially high kidney dose with alpha-emitters [35].

In this congress, the preliminary results of two prospective clinical trials assessing ^68^Ga-PSMA ligands in the context of biochemical failure after radical treatment were reported: in patients with PSA values <2 ng/mL by a team in Bologna and in patients with PSA values <1 ng/ml by a team at Aalborg University Hospital, Denmark. In a single-centre study in patients with PSA values <2 ng/mL, Ceci et al. found a positivity rate of 62% in the first 50 of 100 consecutive patients, with local disease in the prostate bed or pelvic lymph nodes in 15 patients (30%), distant disease in the retroperitoneal lymph nodes or bone in 10 patients (20%), and local and distant disease in 5 patients (10%) [36]. Interestingly, the detection rate was associated with PSA concentration, doubling time (DT) and velocity. In 26 patients with PSA values <1 ng/ml, Nielsen et al. found a detection rate of only 23% (six PET-positive patients), with a mean PSA level of 0.6 ng/ml and a DT of 7 months in PET-positive patients, and a mean PSA level of 0.3 ng/ml and a DT of 18 months in PET-negative patients [37]. These two studies confirm that PSA DT is an important consideration when selecting patients for ^68^Ga-PSMA PET, in particular in patients with low PSA levels (<1 ng/ml).

The use of ^68^Ga-PSMA for primary staging was retrospectively assessed by Uprimny et al. from Innsbruck in 82 patients [38]. PSMA PET was positive in the primary tumour in 93% of patients, with bone foci in ten patients (12%). Patients with a Gleason score of 6 or 7 showed a lower median SUVmax than patients with a Gleason score of >7 and patients with a PSA level <10.0 ng/ml a lower median SUVmax than those with PSA level >10.0 ng/ml, suggesting the value of ^68^Ga-PSMA PET in initial staging in patients with high-risk prostate cancer. A team in Linz confirmed the role of ^68^Ga-PSMA PET in high-risk disease in a series of 61 patients with intermediate-risk (5 patients) and high-risk tumours (56 patients) [39]. At least one malignant lesion was detected in the prostate gland of all patients, and lymph node and bone metastases were detected in 18 and 12 patients, respectively, all with high-risk tumour.

**Fig. 8:**
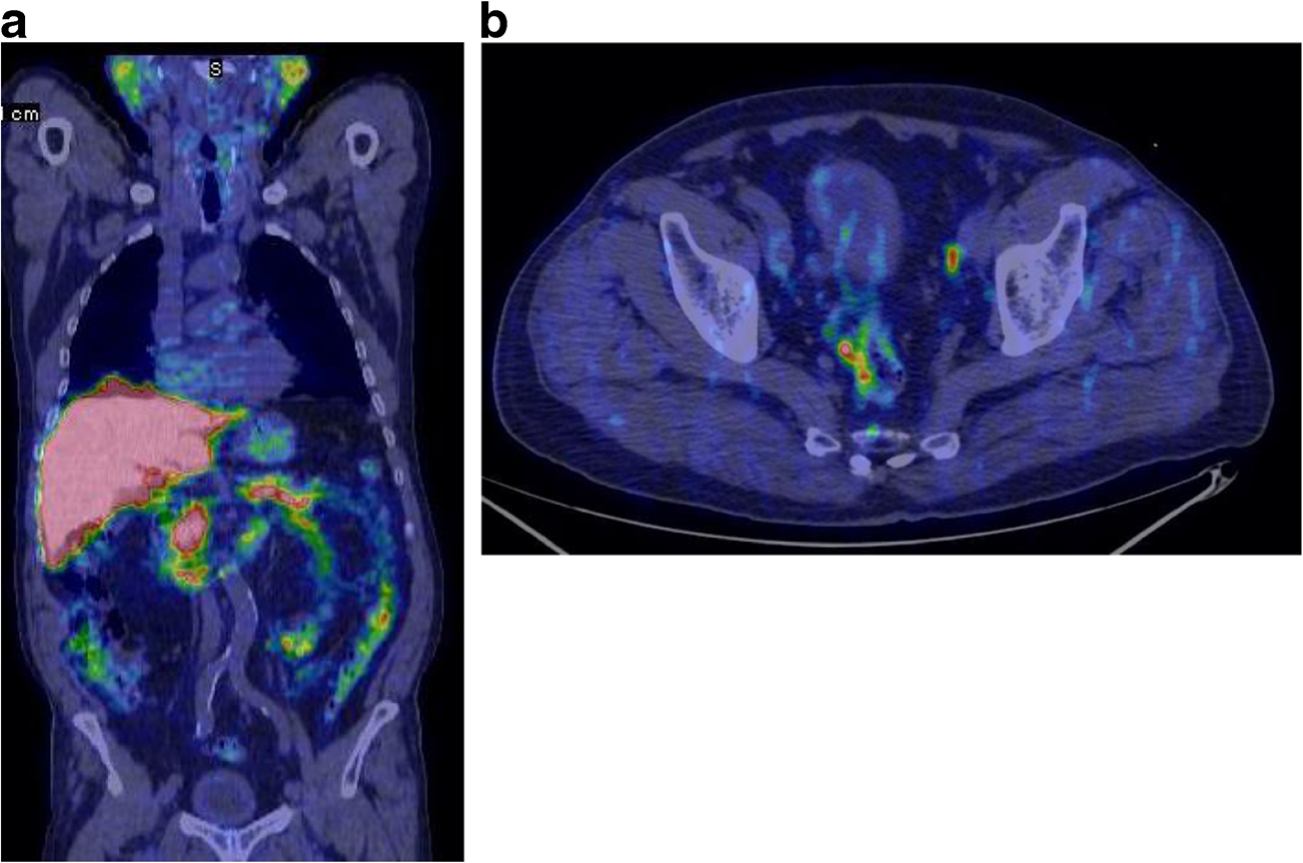
^64^Cu-PSMA PET images in a patient with prostate cancer. An optimal tumour-background ratio was observed at 4 h (**a**). Lymph node uptake was detected in three patients, and was confirmed as tumour by histology (**b**)

Labelling of PSMA ligands with positron-emitting radioisotopes which have longer half-lives than ^68^Ga could be interesting, and Gangemi et al. from Catanzaro, Italy, reported their preliminary experience using ^64^Cu-PSMA PET/CT in 13 patients with intermediate-risk and high-risk prostate cancer before prostatectomy [40]. The administered activity ranged between 259 and 370 MBq and the acquisitions included a static scan of the pelvis at the time of injection and a whole-body scan after 1 and 4 h with 5 min per bed position. ^64^Cu-PSMA PET detected a focal hot spot in the prostate in all patients with SUVmax ranging from 4 to 14. The SUVmax of primary prostatic lesions progressively increased from early to delayed images (30%) with an optimal TBR at 4 h. Lymph node uptake was also detected in three patients, and was confirmed as tumour by histology. A high liver uptake was observed, with SUVmax ranging from 19.5 to 40.5 (Fig. 8). The authors concluded that ^64^Cu-PSMA may be an alternative to ^68^Ga-PSMA because of the high quality of delayed images, and its low urinary excretion and good physical properties.

Radionuclide therapy with PSMA ligands is also a promising approach, and interesting results of PRLT with ^177^Lu were presented at this congress. Ferdinandus et al. assessed predictors of responses to PRLT in 40 patients with distant metastases and progressive disease with hormone refractory and/or chemorefractory disease [41]. ^68^Ga-PSMA PET was recorded 1–2 weeks prior to administration of a mean activity of 6 GBq. A proportion of patients (67.5%) showed a reduction in PSA level 2 months after therapy, with 35% showing a reduction of more than 50%. In a univariate analysis, older age, a lower Gleason score, a lower number of platelets, a lower C-reactive protein level, no need for pain medication and a lower lactate dehydrogenase level reflected a positive impact on response, whereas the response was independent of tumour uptake on ^68^Ga-PSMA PET or previous therapies. Interestingly, Khurshid et al., in a second retrospective analysis in 36 patients, showed the predictive value of textural parameters determined on pretherapeutic ^68^Ga-PSMA PET, demonstrating that the heterogeneity parameters, entropy (*p* = 0.046) and homogeneity (*p* = 0.034), were significantly correlated with PSA response, whereas conventional PET parameters did not predict response [42]. Despite the fact that these data have to be confirmed in larger prospective trials with progression-free survival (PFS) and overall survival (OS) as endpoints, textural analysis on pretherapy PET may help in risk stratification in the context of personalized therapy.

Another interesting question concerns the responses of tumours at different sites to PRLT. Kulkarni et al. from Bad Berka, Germany, assessed the responses of bone and lymph node lesions by ^68^Ga-PSMA PET in 120 patients with progressive metastatic castration-resistant prostate cancer undergoing PRLT with ^177^Lu labelled PSMA inhibitor [43]. The responses of 61 bone and 73 lymph node metastases, also measurable on CT, were analysed after at least two cycles of PRLT using molecular (EORTC) and morphological (RECIST) imaging criteria. PET responses were observed in 53% of bone lesions and 66% of lymph node lesions, the difference in response rates perhaps being due to differences in absorbed dose and radiosensitivity between the tumour sites. Interestingly, CT detected decreases in size in only 6.6% of bone lesions and 39.7% of lymph node lesions, suggesting that ^68^Ga-PSMA PET detected response earlier than morphological imaging. These results need to be confirmed in larger prospective studies.

One of the most important issues regarding PRLT is its potential toxicity that presents, as with peptide receptor radionuclide therapy (PRRT) in NETs, in the form of renal toxicity. The irradiation of the salivary and lacrimal glands during PRLT may also result in xerostomia, a major cause of discomfort for patients [32, 34]. Preloading has been considered to saturate nontumour sites of PSMA uptake. Kletting et al. from Ulm, Germany, reported the results of simulations aiming to determine the effect of cold PSMA-specific peptide administered prior to PRLT on the time-integrated activity coefficients (TIACs) of critical organs and tumour, and thus on the therapeutic index [44]. They showed that the administration of high-affinity (*K*_d_ ≤ 0.1 nM) PSMA-specific peptides 5–30 min before therapy had a substantial effect on the therapeutic index. The optimal preload depended on the affinity of the ligand used. For *K*_d_ = 0.1 nM the model predicted an optimal preload of 128 nmol that would improving the tumour-to-kidney, tumour-to-lacrimal gland and tumour-to-salivary gland TIAC ratios 1.6-fold. These results have to be confirmed by biodistribution studies.

Somatostatin receptor targeting for PRRT and PET was also an important topic at this congress, with the updated results of the NETTER-1 study and also interesting results with innovative therapeutic strategies and dosimetry protocols. NETTER-1 is a major study for the nuclear medicine community because it is the first phase III multicentre, randomized, controlled trial evaluating PRRT [45]. These updated results comparing ^177^Lu-DOTA0-Tyr3-octreotate (Lutathera®; four injections of 7.4 GBq every 8 weeks) with octreotide LAR (60 mg every 4 weeks) in 230 patients with inoperable, progressive, somatostatin receptor-positive grade 1/2 metastatic midgut NETs confirm that PRRT improves PFS, with a median PFS not reached with Lutathera® versus a PFS of 8.4 months with the octreotide control treatment (*p* < 0.0001, HR 0.21). The numbers of patients with a complete or partial response were 18% in the Lutathera group and 3% in the control group (*p* = 0.0008; *n* = 201). Interim analysis also suggested an improvement in OS (14 deaths in the Lutathera group, and 26 in the control group; *p* = 0.0043). Grade 3/4 neutropenia, thrombocytopenia and lymphopenia occurred in 1%, 2% and 9% of patients in the Lutathera group but in none of the patients in the control group.

 Other approaches to PRRT were also considered. McEwan et al. reported their experience in 138 patients with advanced gastrointestinal and pancreatic NETs (GEPNETs) using a novel ^177^Lu-DOTATATE protocol including an induction regimen (four cycles of up to 6.11 GBq per cycle every 10–14 weeks) followed by a maintenance regimen (up to eight cycles of up to 4.07 GBq per cycle every 5.5–10 months) [46]. The median PFS had not been reached at 59.3 months. Transient grade 3 renal and haematological toxicity were reported, but no grade 4 toxicity.

**Fig. 9:**
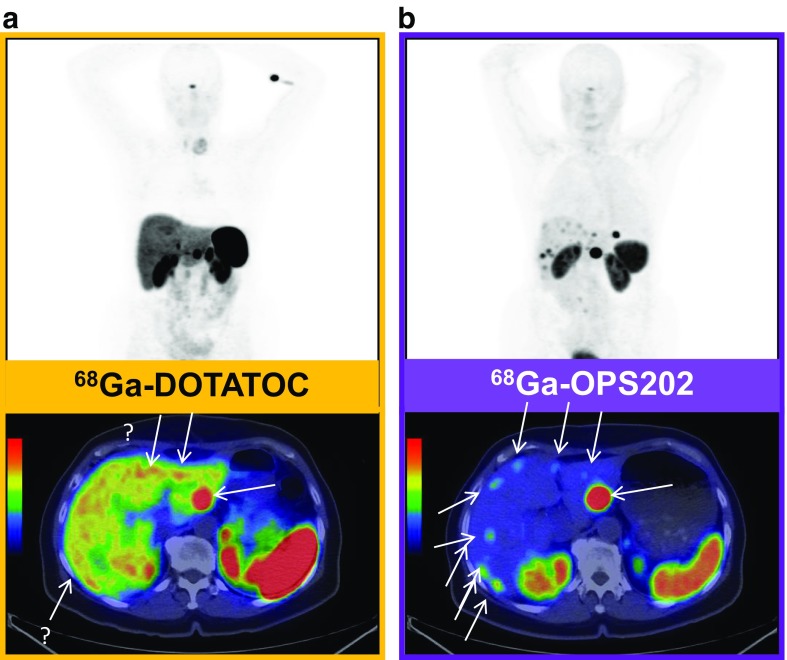
SSTR2 antagonist ^68^Ga-OPS202 for somatostatin receptor PET/CT in gastroenteropancreatic NET. ^68^Ga-OPS202 shows higher sensitivity and image contrast (**b**) than the ^68^Ga-DOTATOC (**a**)

**Fig. 10:**
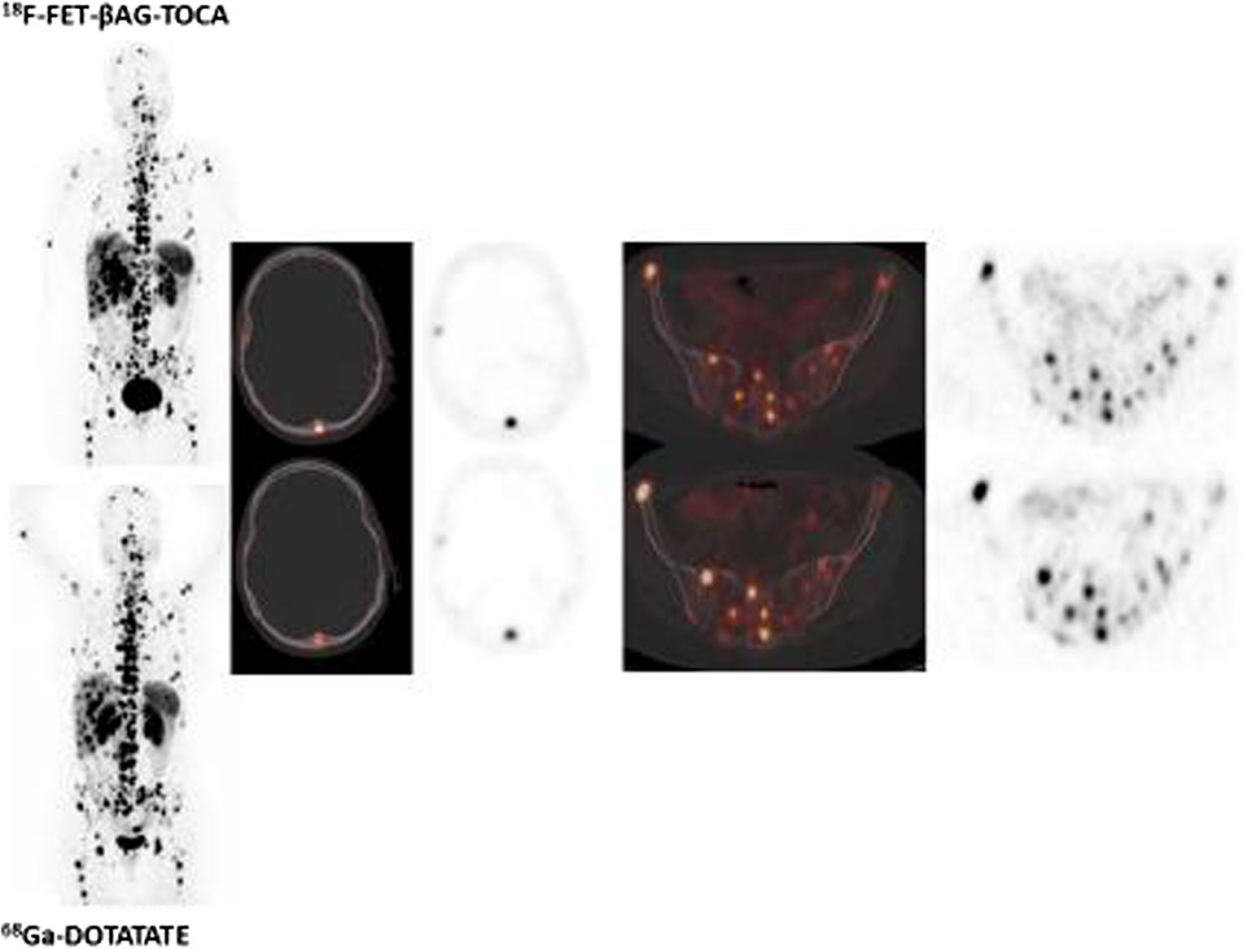
^18^F-Fluoroethyl triazole-labelled [Tyr^3^]octreotate. The authors found non-inferior interim sensitivity and contrast for the new tracer compared with ^68^Ga-DOTATATE

Somatostatin-based PPRT has been in development for some time, so that long-term outcome data have become available. Kunikowska et al. from Poland presented data on the long-term side effects (10-year experience) of tandem peptide radionuclide therapy with ^90^Y/^177^Lu-DOTATATE [47]. In 59 patients with disseminated NET (grade 1 and grade 2, Ki-67 <20%), long-term follow-up revealed a high disease control rate and long PFS with a small number of side effects (no grade 4 and 2% grade 3 renal toxicity; 2% myelodysplastic syndrome but no other grade 3 or 4 haematological toxicity). A cautionary note from Filice et al. from Milan is that baseline ^68^Ga-DOTA-peptide SUVmax is a poor surrogate for the long-term absorbed dose, suggesting the need for routine dosimetry evaluation to achieve a more personalized PRRT [48]. While ^68^Ga-DOTA-peptides remain centre-stage in the staging of patients with NETs, two new imaging methods that could play a role in future staging of this disease were reported. The first was reported by Nicolas et al. from Basel who performed a phase 1/2 trial comparing the SSTR2 antagonist ^68^Ga-OPS202 with the agonist ^68^Ga-DOTATOC for somatostatin receptor PET/CT in patients with gastroenteropancreatic NET [49]. Compared with ^68^Ga-DOTATOC, ^68^Ga-OPS202 showed higher sensitivity (88–94% versus 59% on a lesional basis) and image contrast (4.3–5.3 versus 1.9; Fig. 9). The second method was reported by Dubash et al. from London who presented an interim review of a phase 1/2 trial of a novel fluorinated octreotate analogue (^18^F-fluoroethyl triazole-labelled [Tyr^3^]octreotate) synthesized using ‘click radiochemistry’ in comparison with ^68^Ga-DOTATATE, which showed good sensitivity and contrast for the imaging of NETs [50]. The new tracer can be transported to multiple clinical sites in a similar manner to FDG. The investigators found noninferior interim sensitivity and contrast for the new tracer compared with ^68^Ga-DOTATATE (Fig. 10).

## FDG and radiomics

**Fig. 11:**
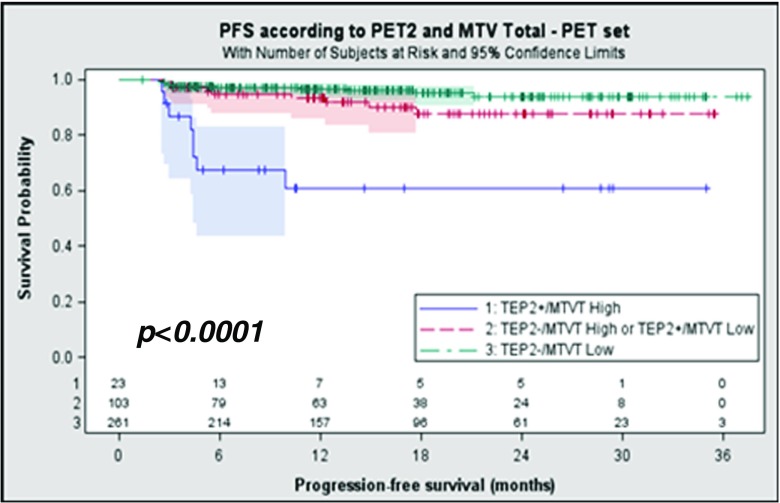
Prognostic value of TMTV in patients with advanced Hodgkin lymphoma enrolled in the AHL2011 trial. The combination of TMTV at baseline and PET positivity after two courses of therapy (PET2/TEP2) allows identification of three groups of patients with significantly different outcomes

More than 50 high-level communications were presented showing the prognostic impact of FDG in oncohaematology, especially using metabolic tumour volume (MTV) or textural indices (TI), indicating that nuclear medicine is on its way to utilizing radiomics for stratified medicine. FDG PET is recommended for therapy evaluation in lymphoma, especially in diffuse large B cell lymphoma and Hodgkin disease, using visual analysis based on the Deauville criteria [51]. Kanoun et al. from Dijon, France, reported the prognostic value of total MTV (TMTV) in 392 patients with advanced Hodgkin lymphoma enrolled in a phase III randomized trial (AHL2011 trial NCT01358747) testing a treatment strategy driven by PET after two courses of treatment (PET2) [52]. The PET2 scans were centrally reviewed and interpreted according to the Deauville criteria, and TMTV was computed on the PET0 scans using the SUVmax threshold of 41%. Using a 350-ml TMTV cut off, with a median follow-up of 16 months, PFS at 2 years was 81% in patients with a high TMTV and 93% in patients with a low TMTV (*p* = 0.0015; HR = 3). Patients positive on PET2 also showed a lower PFS at 2 years than patients negative on PET2 (76% vs. 92%; *p* < 0.0001), and the combination of TMTV and PET2 allowed the identification of three groups of patients with significantly different outcomes, indicating that this approach may help clinicians better tailor therapy (Fig. 11).

**Fig. 12:**
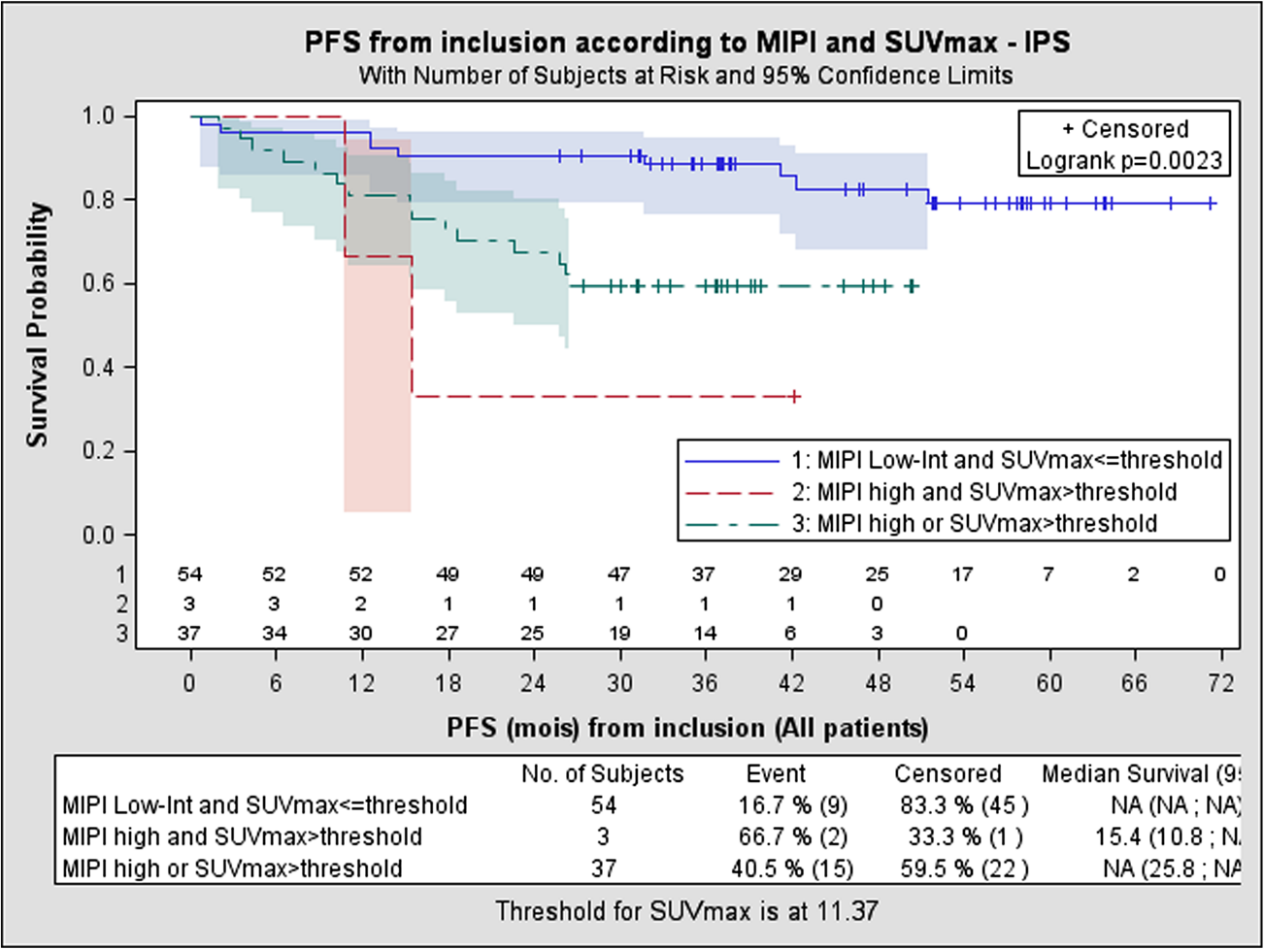
Prognostic value of quantitative indices derived from FDG PET in patients with untreated mantle cell lymphoma included in the LyMa trial. At diagnosis, the prognostic value of SUVmax was reinforced when combined with MIPI, enabling the definition of three prognostic groups

The role and prognostic value of FDG PET in mantle-cell lymphoma is less well defined [51]. Bailly et al. from Nantes, France, assessed the prognostic value of quantitative indices derived from ^18^F-FDG PET in 94 patients with untreated mantle-cell lymphoma included in an ancillary study of the prospective phase III LyMa trial (NCT00921414) [53]. The studied population did not differ from the entire LyMa cohort (*n* = 299). At diagnosis, univariate analysis showed the prognostic value of SUVmax (*p* < 0.001), SUVmean (*p* < 0.001), SUVpeak (*p* < 0.001) and total lesion glycolysis (TLG; *p* = 0.03). The prognostic value of SUVmax was reinforced when combined with the mantle cell lymphoma international prognostic index (MIPI), enabling the definition of three prognostic groups (Fig. 12). Neither whole-body MTV nor whole-body TLG was associated with PFS.

**Fig. 13:**
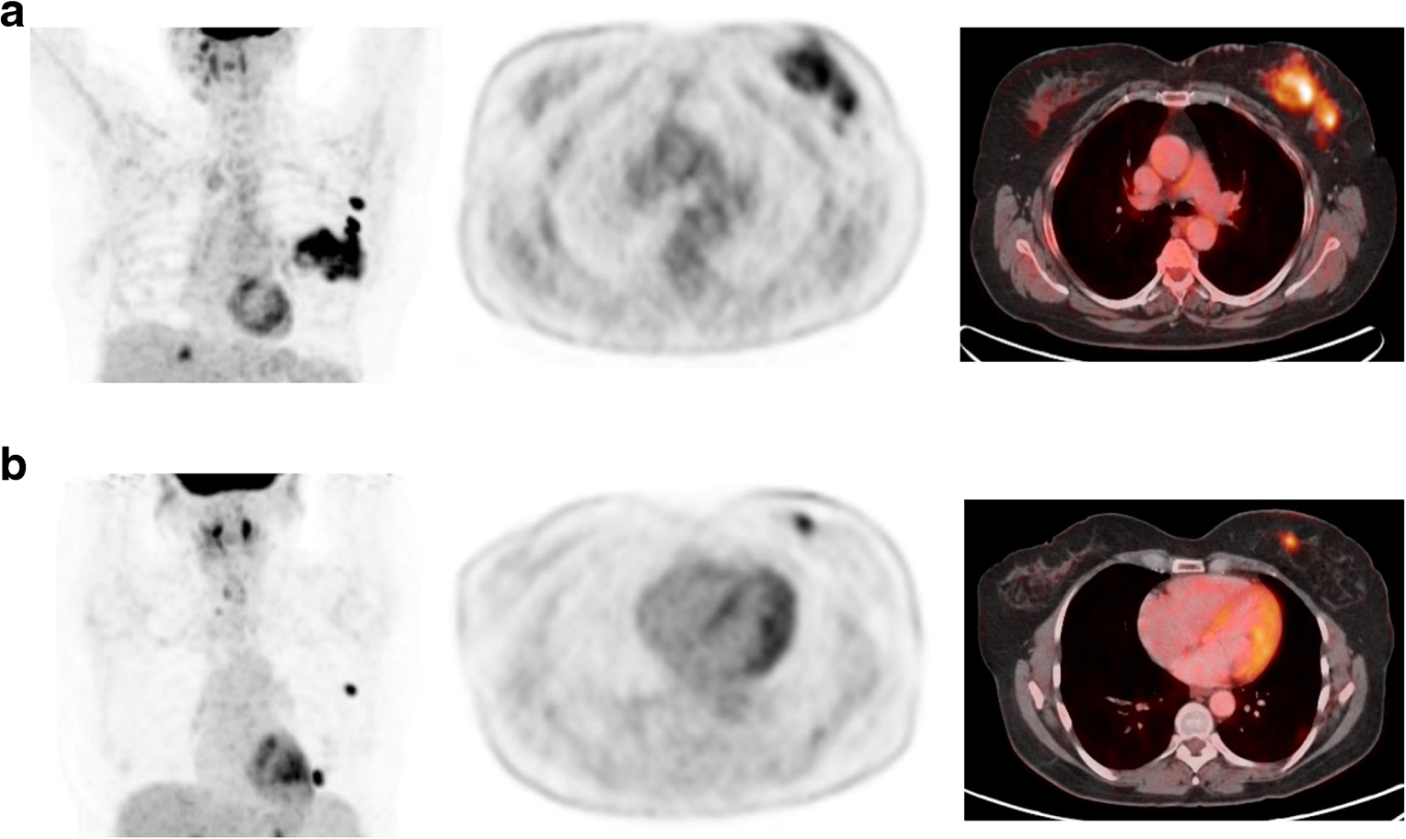
Two patients with breast cancer (**a** and **b**) with similar SUVmax but very different primary tumour MTV and TLG. **a** SUVmax 8.8, MTV 96.5, TLG 396.65; **b** SUVmax 8.5, MTV 2.7, TLG 10.8

The potential utility of quantitative indices derived from ^18^F-FDG PET in breast cancer was also reported by several teams. A group from Madrid retrospectively evaluated the ability of MTV and TLG in comparison with SUVmax and SUVmean in the initial staging of 100 patients with locally advanced breast cancer to predict the recurrence risk and OS [54]. The only parameter related to recurrence was MTV in the primary tumour (*p* = 0.048; Fig. 13). There was also a significant association between MTV measured in the primary tumour and all lesions and OS (31.3 cm^3^ vs. 7.1 cm^3^, *p* = 0.003; and 36.2 cm^3^ vs. 8.7 cm^3^, *p* = 0.006, respectively). Likewise, TLG in the primary tumour and all lesions was higher in patients who died (126.9 g vs. 25.5 g, *p* = 0.007; and 164.6 g vs. 33.8 g, *p* = 0.009). Neither SUVmax nor SUVmean were of prognostic value. Boughdad et al. from Saint-Cloud, France, in collaboration with a team from Orsay, France, investigated whether SUV and TI in breast cancer differed in relation to metastatic status, Ki67 expression, grade and molecular subtype in 122 women who underwent FDG PET/CT for initial staging [55]. Volume, SUV, TLG and homogeneity, entropy, LRE, SRE, LGZE and HGZE were measured in each volume of interest obtained using a 40% threshold and exceeding 1.5 mL in volume. There were no significant differences in SUV and TI between metastatic and non-metastatic patients, whereas significant differences were found for TLG (*p* = 0.028) and volume (*p* < 0.05).

 Interestingly, a team in Milan, Italy, in 44 patients with newly diagnosed breast cancer confirmed the potential prognostic value of TLG, and reported that SUV and TI may also be of value because they showed significant correlations with tumour grade, Ki67 expression and molecular subtype [56]. These different findings suggest the potential value of quantitative indices derived from ^18^F-FDG PET in breast cancer, but the discrepancies among the reports demonstrate that prospective multicentre clinical trials need to be performed in a homogeneous stratified population using a harmonized method to determine the real prognostic value of PET biomarkers. The free software LIFEx developed by the team in Orsay that allows the calculation of indices characterizing tumour heterogeneity should facilitate the collection of data by independent centres [57].

## Non-FDG tracers in clinical use

Promising clinical results were also reported with tracers other than ^18^F-FDG, that allowed better detection or characterization of lesions, especially brain tumours and in haematological diseases. Chiaravalloti et al. from Rome reported the value of posttreatment ^18^F-FDOPA PET/CT in 134 patients with primary brain tumours. Median PFS was 4 months in patients with a PET/CT-positive scan and 25 months in patients with a PET/CT-negative scan (*P* < 0.0001), and median OS was 13 months in patients with a PET/CT-positive scan and 27 months in patients with a PET/CT-negative scan (*P* < 0.0001) [58]. Skvortsova et al. from the Bechtereva Institute of the Human Brain of the Russian Academy of Sciences assessed the diagnostic accuracy of ^11^C-methionine in distinguishing brain tumour recurrence from posttreatment radiation effect (PTRE) in 308 patients with glioma (*n* = 304) or other tumours (*n* = 4) previously treated with multimodal therapy [59]. PET was assessed visually and by calculating an ^11^C-methionine uptake index (UI) as the ratio of the mean lesion uptake to normal cortex uptake. Of lesions with negative tracer uptake (UI < 1.2), 96% represented PTRE or complete metabolic response of glioma, and of lesions with UI > 2.0, 89% represented recurrence. The additional use of MR perfusion allowed the differentiation of lesions with equivocal uptake.

 Lopci et al. from Milan prospectively evaluated the relationship between ^11^C-methionine PET metrics and molecular biomarkers in 109 patients with brain glioma who were candidates for surgery (NCT02518061) [60]. Semiquantitative metrics included SUVmax, SUVratio in relation to normal brain, and MTV. In all patients, tumours were detected with significant differences in SUVmax, SUVratio and MTV in relation to tumour grade (*p* < 0.001). A statistically significant correlation between SUVmax and SUVratio and IDH1 mutation was observed (*p* < 0.001). Relapse or progression was documented in 48 patients (median PFS 8.7 months). Statistically significant correlations were observed between PFS and SUVmax and SUVratio, tumour grade, IDH1 mutation, 1p/19q codeletion and MGMT-promoter methylation.

**Fig. 14:**
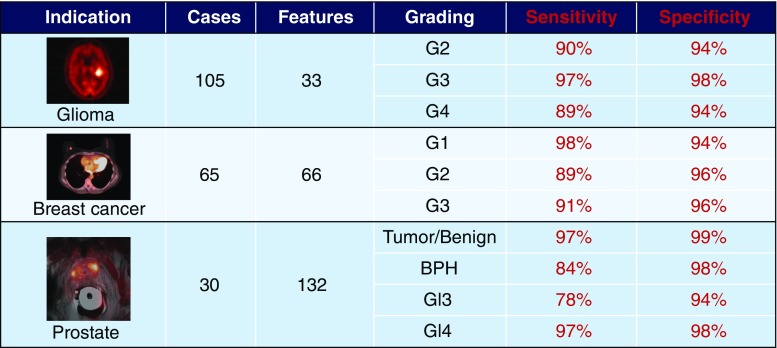
Machine-learning based tumour grading in three different tumour groups

These different reports suggest a PET-derived indices for tumour characterization since their simplicity of interpretation makes their transfer to routine clinical practice likely. Interestingly, a team in Vienna reported the use of computer-support and machine learning algorithms for tumour grading [61–63]. The proposed method was used to grade three different tumour types (glioma, breast cancer and prostate cancer), and high sensitivity and specificity were achieved (Fig. 14). The results indicate that machine learning-based tumour grading has huge potential as a computer-aided decision support method for proper tumour grading and prognosis.

**Fig. 15:**
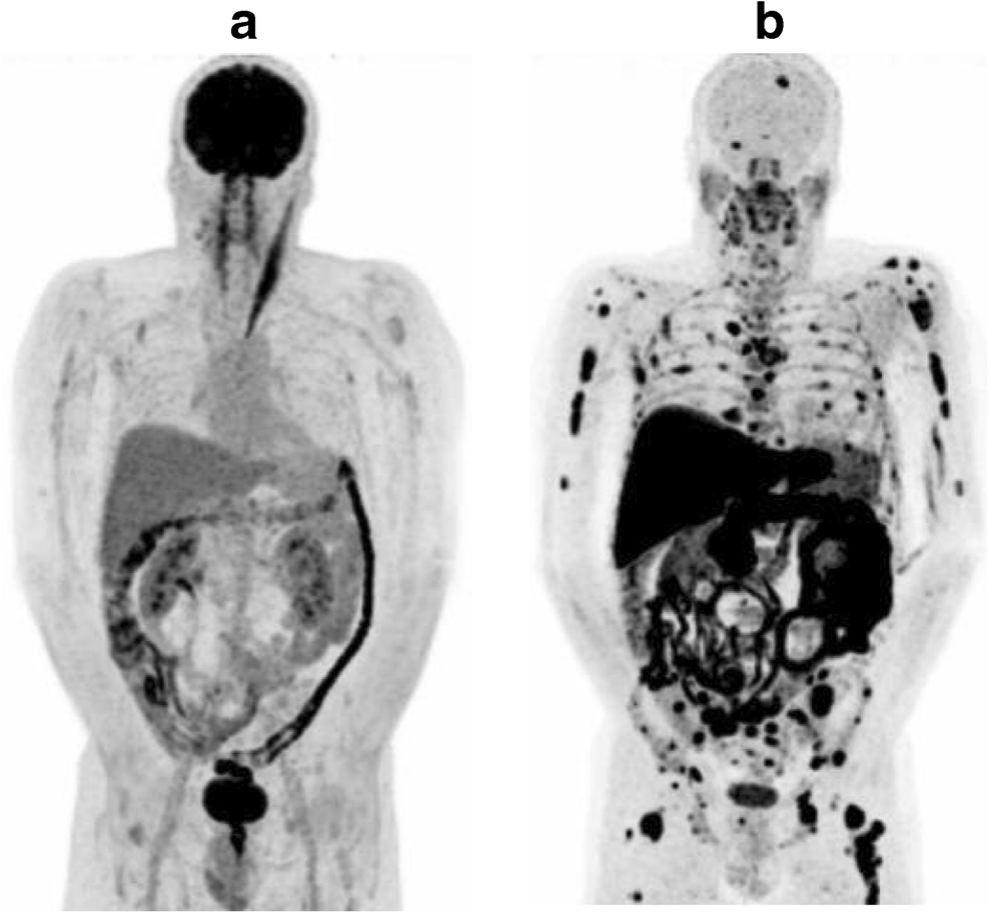
^18^F-FDG (**a**) ^11^C-methionine (**b**) PET/CT images in a patient with multiple myeloma. More lesions are shown by ^11^C-methionine PET/CT (**b**) than by ^18^F-FDG (**a**)

The role of ^11^C-methionine PET/CT has also been assessed in patients with multiple myeloma by a team in Pamplona. More tumour lesions were detected by ^11^C-methionine PET/CT than by ^18^F-FDG (Fig. 15) [64]. Moreover, Zanoni et al. from Bologna reported preliminary data from a single-centre prospective study suggesting that ^18^F-FLT might be complementary to ^18^F-FDG in the detection of lymphoma following a positive or equivocal ^18^F-FDG PET/CT scan at the end of treatment or follow-up [65].

## Radionuclide therapy and dosimetry

Radionuclide therapy is a promising treatment strategy for personalized medicine. The cytotoxic mechanisms by which the agents act are different from those of other therapies, with a limited involvement of cross-resistance and the confounding effect of tumour heterogeneity. Radium-223 dichloride is the latest radiopharmaceutical to have been approved for the treatment of metastatic prostate cancer, having shown an effect on survival in the ALYMPCA phase III study [66]. This trial, together with the NETTER-1 trial discussed above, is potentially of great importance to the nuclear medicine community. Lee et al. from Maidstone and Tunbridge Wells NHS Trust, UK, reported a review of 12 months’ experience with ^223^Ra dichloride for the treatment of patients with prostate cancer and bone metastases [67]. Of 24 patients who completed a 6-month course of intravenous ^223^Ra treatment between March 2015 and March 2016 prospectively reviewed, 80% showed improvement and in 16% pain remained stable with an overall average decrease in pain score of 2.52. Quality of life was more variable, but 75% still responded or remained stable. Follacchio et al. from Rome reported the effects of 301 ^223^Ra cycles in 63 patients [68]. The rates of grade 3/4 thrombocytopenia and anaemia were low (three and six patients, respectively), confirming a favourable haematological toxicity profile. Therapy was effective in relieving pain in 44% of patients, and 44% had stable pain and 12% reported worsening of bone pain. All patients with a high skeletal tumour burden at baseline experienced an improvement in bone pain, suggesting that a palliative rather than a therapeutic effect could prevail in patients with advanced disease.

The key questions in the area of dosimetry relate to (a) whether dosimetry is able to predict efficacy or toxicity, and (b) whether dosimetry during the first course of radiotherapy only is sufficient or repeat measurements with adjustments during multiple courses are necessary. Following earlier work showing that the haematological response during ^177^Lu-DOTATATE treatment is correlated with bone marrow dose [69], Svensson et al. from Lund showed that the radiation exposure of the kidneys affects the haematological response during ^177^Lu-DOTATATE treatment, probably due to impaired renal erythropoietin production [70]. This result indicates that it would be more straightforward if the cumulative kidney dose could be predicted from first cycle of radiotherapy, but a related study by Reijonen et al. from Helsinki did not support this simplification of methodology [71]. In their study, cohort analysis predicted no significant difference in kidney dose per activity between the first cycle and the three successive cycles in the cohort. The scenario was different when patients were ‘individualized’ for analysis (the highest overestimation was 32% and underestimation 22%; the difference was more than 10% for 15 patients and more than 20% for 5 patients) suggesting that dosimetry is needed in every cycle. Generally the outcomes were complex and variable.

## Summary

Overall, we consider that the field of nuclear medicine is making auspicious progress in the development of novel multimodal imaging and its application to diagnosis, staging, therapy selection and therapy monitoring. It is an exciting time to be in this field with the development of new and promising tracers for lesion characterization across disease areas and of several new practice-changing clinical diagnostic tracers and targeted therapies. These developments go to support our view that that nuclear medicine is leading the way in personalizing treatment to patients. We thank the organizers for the opportunity to review the outstanding progress in the field.
